# Effects of Algae Meal Supplementation in Feedlot Lambs with Competent Reticular Groove Reflex on Growth Performance, Carcass Traits and Meat Characteristics

**DOI:** 10.3390/foods10040857

**Published:** 2021-04-15

**Authors:** Nieves Núñez-Sánchez, Carmen Avilés Ramírez, Francisco Peña Blanco, Pilar Gómez-Cortés, Miguel Ángel de la Fuente, Montserrat Vioque Amor, Alberto Horcada Ibáñez, Andrés Luis Martínez Marín

**Affiliations:** 1 Departamento de Producción Animal, Universidad de Córdoba, Ctra. Madrid-Cádiz km 396, 14071 Córdoba, Spain; pa2nusan@uco.es (N.N.-S.); pa1peblf@uco.es (F.P.B.); 2 Departamento de Bromatología y Tecnología de los Alimentos, Universidad de Córdoba, Ctra. Madrid-Cádiz km 396, 14071 Córdoba, Spain; v92avrac@uco.es (C.A.R.); bt1viamm@uco.es (M.V.A.); 3 Instituto de Investigación en Ciencias de la Alimentación (CIAL, CSIC-UAM), Universidad Autónoma de Madrid, Nicolás Cabrera, 9, 28049 Madrid, Spain; p.g.cortes@csic.es (P.G.-C.); mafl@if.csic.es (M.Á.d.l.F.); 4 Departamento de Agronomía, Escuela Técnica Superior de Ingeniería Agronómica, Universidad de Sevilla, 41013 Sevilla, Spain; albertohi@us.es

**Keywords:** marine algae, lambs, performance, meat quality

## Abstract

There is growing interest in increasing omega-3 fatty acid (FA) contents in ruminant meat by means of dietary manipulation, but the effects of such manipulation on productive results and meat quality need to be ascertained. The aim of the present study was to assess the effects of supplementing lambs with competent reticular groove reflex (RGR) with marine algae as a source of omega-3 fatty acids on growth performance, carcass traits, and meat quality characteristics. Forty-eight feedlot lambs were distributed into three equal groups: the control group neither consumed marine algae nor had competent RGR, the second group received daily 2.5% of algae meal mixed in the concentrate, and the last group consumed the same amount of algae meal, but emulsified in a milk replacer and bottle-fed. Lambs in the second and third groups had competent RGR. There were not any negative effects on performance, carcass or meat quality parameters with algae supplementation. However, the results of the oxidative stability parameters were not conclusive. Ageing for 6 days improved meat tenderness and color, and increased lipid oxidation. In conclusion, algae meal inclusion in the diet of fattening lambs with competent RGR has no detrimental effects on animal performance, carcass traits or meat quality characteristics.

## 1. Introduction

Lamb meat is widely consumed in some geographical areas, for instance in Mediterranean countries, but has a non-healthy nutritional image, mostly due to the general idea of having high levels of saturated fatty acids (FA), variable contents of trans-fat, and low levels of omega-3 polyunsaturated FA [1]. However, the quality of lamb meat and its FA profile is closely related to the feeding conditions of the animals. The intramuscular fat (IMF) of lambs reared under intensive feeding conditions is characterized by high levels of saturated and omega-6 FA, and a low amount of omega-3 FA [2,3,4,5]. In contrast, meat from grass-fed lambs has shown a more desirable FA composition, with lower contents of saturated FA and higher levels of omega-3 FA [6].

In the last decade, there has been growing interest in finding appropriate and natural ways to manipulate IMF composition, with grazing being one of the best alternatives. However, in regions where fattening on pasture is not feasible for climatic reasons, other approaches to increase omega-3 FA have been evaluated, such as adding plant-derived oils rich in omega-3 FA [7,8,9]. Dietary marine algae have been shown to improve meat nutritional value in lambs [8,10,11,12,13] due to their high eicosapentaenoic (EPA), docosapentaenoic (DPA) and docosahexaenoic (DHA) FA contents. Very recently, our group has reported that the dietary inclusion of marine algae at the level of 2.5% enhanced omega-3 FA contents in lamb meat with competent reticular groove reflex (RGR), with the increase being significantly higher when the marine algae was bottle-fed in comparison to the same amount of algae meal mixed in the concentrate [14]. Fostering the RGR of the newborn animal into adulthood and using it to include emulsified lipid sources into the abomasum, bypassing the rumen, has been demonstrated as a fruitful strategy to enhance the healthy unsaturated FA presence in ruminant milk and meat [14,15].

Marine algae supplementation may have undesirable effects on growth performance, carcass traits and meat quality characteristics [16], such as a decrease in average daily gain and feed intake, and thus an extension of the fattening period [10,17]. Moreover, the high content of polyunsaturated FA in the IMF from lambs fed marine algae increases its peroxidability index [14], which could lead to meat quality degradation during storage [18]. Changes occurring in the meat micro- and ultra-structure during the aging process are associated with favorable modifications in its meat tenderization and water-holding capacity. However, as stated previously, the oxidation of lipid components, as well as the destabilization of meat color, can negatively affect the meat’s final value [19]. Again, marine algae are a good source of antioxidants. Most researchers consider polyphenolic compounds (i.e., phenolic and cinnamic acids, phlorotannins, and bromophenols) among the main factors responsible for such antioxidant properties [20].

The farm to fork strategy includes hygienic, compositional, nutritional, sensory, and technological quality characteristics in the general concept of meat quality throughout the food chain, in order to obtain high-quality products [21].

The aim of this study was to assess the effects of supplementing lambs with competent RGR with marine algae as a source of omega-3 FA on growth performance, carcass traits and meat quality characteristics. 

## 2. Materials and Methods

### 2.1. Experimental Design and Diets

This experiment was carried out on the premises of the Animal Production building of the University of Córdoba (Spain). Details about the experimental design and diets have recently been published [14]. Briefly, a total of 48 male Manchega-breed lambs at 42 days of age were weighed (11.6 ± 1.67 kg) and assigned in pairs of similar body weights to 1 of 24 adjacent pens (1.40 m^2^ raised slatted floor cages with individual troughs for feed and water, within an environmental controlled room). Pens were distributed in 8 blocks according to average body weight and allocated randomly to one of the three treatments (16 animals per treatment): (i) the control group, which consisted of a typical pelleted concentrate without algae meal supplementation (NOALG), (ii) the algae meal in concentrate group, which received the same concentrate as NOALG but mixed with 2.5 % algae meal (*Aurantiochytrium limacinum*; Forplus, Alltech Spain, Guadalajara, Spain) plus 250 mL daily of milk replacer in a single feeding (ALGCON), and (iii) the algae meal in milk replacer group, which received the same concentrate as NOALG plus 250 mL daily of milk replacer supplemented with algae meal in a single feeding (ALGMILK). The concentrate was composed by 70% cereals (barley, maize and wheat, 40:20:10), 20% soybean meal, 6% wheat bran and 4% minerals and vitamins, so its calculated composition was 16% crude protein and 11.0 MJ/kg metabolizable energy, as fed. In the ALGCON and ALGMILK diets, milk replacer provided an extra 0.89 MJ of metabolizable energy per day, and algae meal a total of 0.75 MJ/kg of concentrate. Besides this, these diets caused an increase in the crude protein intake of 10 g/d with the milk replacer and 3 g/kg of concentrate with the algae meal. The ALGCON and ALGMILK treatments provided ~584 mg of omega-3 FA per 100 g of concentrate consumed. Lambs in the ALGCON and ALGMILK groups had competent RGR. All animals had free access to wheat straw and fresh water throughout the whole experimental period (49 days on fattening).

### 2.2. Sampling and Analysis

The average body weight per pen was recorded weekly during the experiment, in the morning before feeding. The average daily feed intake (ADFI, kg/day) per pen was calculated daily by weighing each day the amount of concentrate offered and subtracting the amount of feed refusals found in the feeders the following morning. Besides this, average daily gain (ADG, kg/day) and feed conversion ratio (FCR, feed to gain kg/kg) were calculated for each pen. 

When the average body weight of the set of lambs reached ~25 kg (in the range of the commercial slaughter weight for this type of lambs), the animals were tagged to track their carcasses, and transported from the Animal Production facilities at the University of Cordoba to a commercial slaughterhouse (COVAP, Pozoblanco, Córdoba, Spain), located ~90 km (~1.0 h) away, in a vehicle adequately conditioned. Then, the animals were placed in pens (8 lambs per pen) and remained there for approximately 14 h, with free access to water but not feed. After lairage, lambs were stunned, slaughtered, and dressed.

The carcasses of the animals were weighed in the first 45 min after slaughter (hot carcass weight, HCW). The dressing was calculated as the ratio of HCW to final body weight and expressed as a percentage. Lamb carcasses were classified according to their weight as class A (<7 kg), class B (7.1 to 10 kg), and class C (10.1 to 13 kg). A trained and experienced technician from the slaughterhouse visually graded carcass fatness considering the size of the kidney and pelvic fat deposits (types 1, low fat; 2, medium fat; 3, high fat) and muscle color (pale pink, pink, red) [22]. 

After 2 h at room temperature, all carcasses were chilled at 4.0 °C for 24 h in a commercial chiller and transferred to the Animal Production laboratory without disrupting the cold chain. The left shoulders were weighed and dissected into muscle, bone and fat (subcutaneous, pre-scapular and intermuscular) and the remaining tissues (major blood vessels, ligaments, tendons and fascias). Each fraction was weighed, and the results were expressed as the percentage of total shoulder weight to provide an estimate of the carcass composition. At 24 h post-mortem, the *Longissimus thoracis* muscle (T6 to T13 vertebrae) from each left carcasses was removed and divided into three pieces (T6, T7 to T12, and T13 vertebrae), which were further used for the assessment of meat quality (pH, color, drip and cooking losses, and Warner–Bratzler shear force) and parameters related to meat oxidative stability. The *Longissimus thoracis* muscle from the right carcass was vacuum-packed, aged in a refrigerated chamber at 2–4 °C in the dark for 6 days (7 d postmortem), and further used for the same determinations.

The methodology of the measurements is described in detail in Avilés et al. [2]. Briefly, the drip loss (DL) of each sample was expressed as the percentage of weight loss of a sample hanging at 4° for 24 h, related to the initial weight. The pH was measured by inserting the glass electrode of a portable pH-meter (Crison^®^ PH25, Hach Lange, Barcelona, Spain) approximately 1 cm into the *Longissimus thoracis* muscle between the T11 and T12 junction sites. Meat color was measured on the freshly cut surface of the middle section of the T12 vertebra, after 30 min of blooming at room temperature in the dark. A CM-2600d hand-held spectrophotometer (D65 illuminant, 8 mm diameter aperture, 10° standard observer, 8° viewing angle; Minolta Inc., Osaka, Japan) was used for color determination, according to the CIE system (CIE, 1986). Three measurements of color coordinates, expressed as L* (lightness), a* (redness) and b* (yellowness), were performed, and the average was used for the calculation of color saturation (C*) and hue (h°). For the determination of cooking loss (CL) and Warner–Braztler shear force (WBSF), the samples were weighed, cooked in a plastic bag in a water bath at 75 °C until the temperature in the center of the sample reached 70 °C (monitored by a HI 98509 Checktemp^®^ Pocket Thermometer, Hanna Instruments, Guipuzcoa, Spain), and then cooled at room temperature for 30 min, blotted dry, and weighed again. CL was expressed as the percentage of weight loss related to the initial weight. WBSF values, expressed in kg/cm^2^, were taken for muscle cores of 1 cm^2^ cross-sections using a texture analyzer (TA.TXT-2, Stable Micro Systems, Surrey, UK) equipped with a Warner–Bratzler shearing device (Mitutoyo series 500, Mitutoyo Corporation, Aurora, IL, USA). A total of 10 measurements was performed in each meat sample, and the average was provided as the WBSF value of the sample. The extent of lipid oxidation was assessed by measuring the thiobarbituric acid reacting substances (TBARS) using the method of Tarladgis et al. [23], as described in Avilés et al. [2]. The 1,1,3,3-tetra-ethoxypropane (TEP) standard curve was used for calculating the TBARS concentration and the results were expressed as mg of MDA kg^−1^ of meat. The antioxidant capacity of the meat was determined through the 2,2-diphenylpicrylhydrazyl (DPPH) radical scavenging capacity assay. The meat hydrophilic and lipophilic extracts were obtained following the method described by Folch et al. [24]. The determination of the free radical scavenging activity of the hydrophilic and lipophilic extracts of the meat samples (DPPH water and DPPH fat, respectively) was carried out following the procedure described by Brand-Williams et al. [25]. The absorbance values in both hydrophilic and lipophilic extracts were expressed as % radical scavenging activity (RSA). Total polyphenols content was determined with the Folin–Ciocalteu reagent according to the procedure described by Singleton [26]. Total phenolic compounds were quantified using a gallic acid reference standard, and the results were expressed as mg of gallic acid equivalents (GAE)/100 g of meat.

### 2.3. Statistical Analysis

SAS UE 3.8 software (SAS Institute Inc., Cary, NC, USA) was used to perform the statistical analyses. Statistical significance was declared at *p* < 0.05. Productive and carcass traits, except for grading, were analyzed with the MIXED procedure. The statistical model included the treatment as fixed effect and the pen nested within the treatment as the random effect. When the fixed effect was significant, differences between least squares means were assessed by Tukeys’s test. A repeated measurements analysis of meat data was carried out with the MIXED procedure. The statistical model included the fixed effects of treatment, ageing time, and their interaction; the repeated effect was ageing time; the subject of the repeated measurements was the animal nested within the treatment and the pen. When the fixed effects of the repeated measurement model were significant, differences between least squares means were assessed by paired *t*-test.

## 3. Results

### 3.1. Growth Performance and Carcass Traits

Growth performance was not affected (*p* > 0.05) by the addition of algae meal to the experimental rations (Table 1). According to HCW, 22% of the carcasses were classified as class A and 78% as class B, whereas according to fatness most carcasses were within type 2 (60% in comparison to 34% and 6% in types 3 and 1, respectively). The subjective carcass color was pale pink and pink in 65 and 35% of the carcasses, respectively. The lowest HCW and dressing percentage were observed (*p* < 0.05) in ALGMILK, while ALGCON treatment showed the highest values (Table 1). ALGCON treatment had lower and higher percentages of carcass muscle and fat, respectively, than NOALG (*p* < 0.05), while ALGMILK showed intermediate values.

### 3.2. Meat Quality Characteristics and Oxidative Stability

The meat quality characteristics and oxidative stability results are shown in Table 2. Neither the treatment nor the ageing time affected meat pH or DL (*p* > 0.05). CL and WBSF parameters were not modified by the treatments (*p* > 0.05), but were reduced by ageing time (*p* < 0.05). The addition of algae meal to the diet did not affect meat color (*p* > 0.05), apart from the b* coordinate, which was lower (*p* < 0.05) in NOALG. On the contrary, ageing time influenced (*p* < 0.05) the color coordinates, causing a significant increase in L* and a*, as well as a decrease in the b*, C* and h° parameters in all experimental groups.

The presence of algae meal in the diet tended to increase TBARS (*p* = 0.06), while ageing increased (*p* < 0.05) TBARS. At day 1 of ageing, the DPPH fat was higher (*p* < 0.05) in the ALGCON than in the ALGMILK treatment, with an intermediate value for NOALG, but the differences disappeared (*p* > 0.05) at day 6 of ageing. DPPH water did not differ (*p* > 0.05) among treatments, but decreased (*p* < 0.05) with ageing time. As regards the polyphenol content in the diets, the algae meal supplementation supplied 11.8 mg GAE/100 g, while the amount of GAE/100 g in the NOALG diet was 10.3 mg. The total polyphenols contents in meat were not affected by treatments or ageing. 

Regarding oxidative stability, it is worth mentioning that marine algae increased total SFA levels in the ALGCON and ALGMILK treatments (40.3 and 40.2%, respectively, vs. 36.9% of total FA in the NOALG treatment), did not affect total MUFA (38.15, 37.86, and 36.60% of total FA in the NOALG, ALGCON, and ALGMILK treatments, respectively), and raised total omega-3 FA (1.45, 3.94 and 4.96 % of total FA in the NOALG, ALGCON, and ALGMILK treatments, respectively). The increases in the levels of omega-3 FA, EPA, DPA, and, most significantly, DHA, were higher when the algae meal was bottle-fed via RGR, in comparison to algae meal mixed in the concentrate [14].

## 4. Discussion

### 4.1. Growth Performance and Carcass Characteristics

The slaughter weights of lambs fed different experimental diets were within the range of 22–30 kg BW, as established in the European Regulation for “Cordero Manchego” protected geographical indication [27]. The average feed intake, average daily gain and feed conversion ratio values from the present study were more favorable than those obtained previously by Avilés et al. [2] using lambs from the same genetic background, with similar ages and slaughter weights, raised in on-farm conditions. 

The overall performance of the lambs was not affected by the addition of algae meal to the diet in the present study, which is in agreement with previous research, wherein the diets of fattening lambs were supplemented with *Aurantiochytrium limacinum*, *Arthrospira platensis*, and *Isochiris* sp. [8,11,28,29]. However, the results in the literature regarding algae meal supplementation to the diet of lambs are somehow contradictory. Other researchers found that lambs fed diets containing *Aurantiochytrium limacinum* algae had slower growth rates at 1.7% of inclusion [7], or lower average feed intakes and daily gains at 2 to 6% of inclusion [10,13,17], in comparison with the control lambs. In contrast, increased average feed intake, daily gain and final body weight in lambs fed *Arthrospira platensis* algae meal at 1 g/10 kg BW/d were also reported [30]. These discrepancies could be mainly attributed to the type and amount of algae used, its palatability, the trial duration, and/or the weights and ages of the lambs following algae treatments.

As in the present study, previous research did not find differences in HCW and dressing percentage between control and algae-added diets [7,8,11,13,29]. Nonetheless, the means of administration of the algae meal showed a marked influence on HCW and dressing, being significantly higher when algae meal was fed in the concentrate (ALGCON) and reduced when the same amount of supplement was fed in the milk replacer (ALGMILK). Regarding the carcass composition, the present research is in agreement with previous results [2], and the reported values are within the normal ranges for this type of meat. On average, the algae meal treatments showed ~12% more carcass fat than NOALG, which might be related to a higher percentage of fat in the former’s daily gain due to the extra energy consumed via milk replacer and algae meal [31]. 

### 4.2. Meat Quality Characteristics and Oxidative Stability

Ultimate pH (24 h after slaughter) and its fall rate are widely used to evaluate raw meat quality, due to its strong relationship with meat quality characteristics such as color, water-holding capacity and tenderness [32,33]. In the present study, neither treatments nor ageing time affected meat pH (Table 2). The ultimate pH ranged from 5.70 to 5.77 (Table 2), values typically observed in non-stressed sheep at the time of slaughter [34,35]. The observed pH values (Table 2) were within the expected range for this type of lamb meat, and were in agreement with those reported by other authors [2,8,17,29]. The lack of changes in meat pH due to ageing is in line with previous studies [19,36,37]. 

DL was not influenced by the addition of algae meal to the diet, and remained stable throughout ageing (Table 2). The DL values measured at day 1 were in agreement with those reported by other authors [8,13]. Regarding aged samples, the DL values were slightly higher than those shown by Avilés et al. [2] and Vergara et al. [35,37] in Manchega lambs.

Treatments had no effect on CL, or loss of water with cooking. The CL values (Table 2) differed from those obtained by other authors [2,38,39]. The slaughter weight, fatness, pH, cooking procedure and cooling time, among others, are factors to which these differences could be attributed [40]. The numerical trend of lower CL in day 7 samples matched with the observed trend of carcass fat percentage, suggesting that as fat increased, the aged samples were protected from losing water [41,42]. Conversely, Hopkins et al. [29] found that the CL values were significantly higher in algae-fed lambs, but those animals had no increased subcutaneous fat depth.

Meat tenderness, measured as WBSF, was not affected by the inclusion of algae meal in the diet, neither in fresh nor in aged meat samples, which is in agreement with Valença et al. [13] and Hopkins et al. [29]. The average WBSF value at day 1 was greater than the threshold (5 kg/cm^2^) reported by Shorthose et al. [43] to classify lamb meat into tender or tough, while, after 6 days of ageing, it was similar to that value. The maximum shear force recorded in the present study was similar to that obtained by Avilés et al. [2], Blanco et al. [38] and Linares et al. [44]. As expected, ageing time caused a significant decrease in WBSF, as previously described [45]. 

The meat color coordinates L*, a*, C* and h° were not affected by the algae meal treatments (Table 2), in agreement with previous research [8,12,13,29]. Although b* was higher on average in the algae meal treatments, it did not affect h° and C* values, and thus did not alter the perception of color (from red to yellow) or its vividness, respectively [46]. Desirable changes in color indices were observed during ageing in the present study (i.e., higher L* and a* values and lower b* and h° values), as observed by other authors [39]. 

Algae meal in the diet tended to increase meat oxidation measured as TBARS in day1 samples (Table 2), whereas ageing significantly raised TBARS regardless of the treatment. All TBARS values were lower than the acceptability limit of 2 mg MDA kg^−1^ muscle to detect rancidity or oxidized flavors in cooked lamb meat by consumers [18,47,48]. Previous research has shown that algae meal supplementation increases meat lipid oxidation levels [8,12,13,17], which can be attributed to the higher long chain omega-3 polyunsaturated FA content in the IMF of algae meal-supplemented lambs [14]. 

Meat antioxidant activity was evaluated through the DPPH radical scavenging capacity of both lipophilic (DPPH fat) and hydrophilic (DPPH water) extracts (Table 2). Day1 ALGMILK samples, which contained 5 g of omega-3 FA/100 g of total fat [14], showed less DPPH fat than those from ALGCON treatment, which contained 20% less omega-3 FA. This difference would be mainly related to the need to eliminate the free radicals generated by the extra omega-3 FA in ALGMILK samples [18]. Ageing time decreased both the DPPH fat and the DPPH water of NOALG and ALGCON samples, but a similar antioxidant capacity was observed in all aged meats. 

The meat’s total polyphenols contents were not affected by dietary treatments (Table 2), which is in agreement with Muiño et al. [49], who did not observe differences after the supplementation of sheep diets with red wine polyphenols (900 mg of red wine extract/kg of feed). On the contrary, Luciano et al. [50] reported higher concentrations of polyphenols in the meat of lambs fed a diet enriched with tannins for 60 days (8.96% quebracho supplement rich in proanthocyanidins), compared to control lambs. Although algae are recognized as a good source of polyphenols, it may have been the case that the amounts of marine algae included in the current experimental diets or the length of supplementation were not sufficient to trigger any significant response [51].

## 5. Conclusions

The results of this study show that the addition of 2.5% marine algae to the diet of fattening lambs with competent RGR, either mixed in the concentrate or bottle-fed, does not have any negative effect on growth performance, carcass characteristics, meat quality, or, for the most part, oxidative stability. This “from farm to fork” strategy, intended to increase the levels of omega-3 polyunsaturated FA in meat, would be of great interest to the improvement of lamb meat from a nutritional point of view, without affecting animal performance or other meat quality parameters.

## Figures and Tables

**Table 1:** Growth performance and carcass characteristics of feedlot lambs fed a conventional diet alone (NOALG) or feedlot lambs with competent reticular groove reflex fed the same diet supplemented with 2.5% of algae meal, either mixed in the concentrate (ALGCON) or in the milk replacer (ALGMILK).

	DIET				
Parameters ^1^	NOALG	ALGCON	ALGMILK	SEM	*p*
Initial body weight (kg)	11.4	11.6	11.8	0.24	0.86
Final body weight (kg)	25.0	25.7	25.4	0.31	0.78
Average feed intake (g)	825	801	805	6.0	0.44
Average daily gain (g)	326	335	324	4.2	0.33
Feed conversion ratio (kg/kg)	2.54	2.43	2.49	0.030	0.13
Hot carcass weight (kg)	10.5 ^ab^	11.4 ^a^	10.4 ^b^	0.16	<0.05
Dressing ^2^ (%)	42.2 ^ab^	44.6^a^	40.9 ^b^	0.60	<0.05
Carcass composition ^3^:					
Muscle (%)	60.8 ^a^	58.0 ^b^	59.8 ^ab^	0.38	<0.05
Fat (%)	14.5 ^b^	17.2 ^a^	15.2 ^ab^	0.39	<0.05
Bone (%)	24.8	24.8	24.9	0.19	0.95

^1^ Within each treatment, 8 pens were used for determination of growth performance (2 animals per pen) and 16 animals were used for determination of carcass traits. ^2^ Calculated as hot carcass weight/final body weight. ^3^ Estimated by shoulder dissection. SEM: standard error of the mean. Means with different superscripts between treatments are significantly different (*p* < 0.05).

**Table 2:** Quality characteristics and oxidative stability of meat samples from feedlot lambs fed a conventional diet alone (NOALG) or from feedlot lambs with competent reticular groove reflex fed the same diet supplemented with 2.5% of algae meal, either mixed in the concentrate (ALGCON) or in the milk replacer (ALGMILK).

Parameters ^1^	Ageing Time (A)	Diet (D)			SEM	*p*		
		NOALG	ALGCON	ALGMILK		D	A	D × A
Meat characteristics								
pH_24_	1	5.77	5.70	5.73	0.01	0.21	0.19	0.87
	7	5.78	5.72	5.74				
Drip Loss (%)	1	1.93	1.74	1.63	0.06	0.46	0.24	0.06
	7	1.49	1.86	1.59				
Cooking Loss (%)	1	21.5	22.7	20.6	0.96	0.33	<0.01	0.19
	7	19.9	12.7	15.0				
WBSF (kg/cm^2^)	1	7.85	7.28	7.28	0.21	0.97	<0.001	0.07
	7	4.85	5.18	5.38				
L*	1	37.4	38.7	38.8	0.34	0.30	<0.001	0.87
	7	41.6	42.7	42.6				
a*	1	6.27	5.67	6.06	0.20	0.97	<0.001	0.09
	7	7.87	8.55	8.29				
b*	1	16.4	16.7	16.7	0.32	<0.05	<0.001	0.18
	7	10.0	11.1	11.1				
C*	1	17.6	17.7	17.7	0.24	0.20	<0.001	0.13
	7	13.0	14.2	14.0				
h°	1	69.3	71.5	70.2	1.09	0.54	<0.001	0.44
	7	50.7	51.6	52.5				
Oxidative stability								
TBARS (mg MDA/kg)	1	0.23	0.67	0.62	0.05	0.06	<0.001	0.24
	7	0.97	1.06	1.14				
DPPH fat (%)	1	24.2 ^AB,a^	29.0 ^A,a^	17.5 ^B^	1.03	<0.05	<0.001	<0.001
	7	14.6 ^b^	15.0 ^b^	14.4				
DPPH water (%)	1	6.28	6.76	6.63	0.31	0.78	<0.01	0.96
	7	4.20	4.58	4.81				
Polyphenols (mg GAE/100 g)	1	3.72	3.93	4.34	0.07	0.19	0.92	0.21
	7	4.10	3.91	4.03				

^1^ In each time within each treatment, 16 samples were analyzed for each parameter. SEM: standard error of the mean. L*: lightness; a*: redness; b*: yellowness; C*: chroma; h°: hue; WBSF: Warner–Braztler shear force; TBARS: thiobarbituric acid reactive substances; DPPH fat: 2-2 diphenyl picryl hydrazyl in fat extract; DPPH water: 2-2 diphenyl picryl hydrazyl in aqueous extract. Means with different superscript letters between treatments (capital) or ageing times (lowercase) are significantly different.

## Data Availability

The data presented in this study are available in the article.
